# Serum cystatin C as a biomarker in non-Hodgkin lymphoma and its relationship with paraoxonase 1 activity

**DOI:** 10.5937/jomb0-61502

**Published:** 2026-04-15

**Authors:** Bosa Mirjanić-Azarić, Smiljana Mijić, Zana Radić-Savić, Siniša Stanković, Dragana Malčić-Zanić, Egeljić Nataša Mihailović, Đorđe Stojisavljević, Bojana Ivetić, Nataša Bogavac-Stanojević

**Affiliations:** 1 University of Banja Luka, Faculty of Medicine, Department of Medical Biochemistry, Banja Luka, The Republic of Srpska, Bosnia and Herzegovina; 2 Institute of Laboratory Diagnostics, University Clinical Centre of the Republic of Srpska, Banja Luka, Republic of Srpska, Bosnia and Herzegovina; 3 Aqualab laboratory, Banja Luka, The Republic of Srpska, Bosnia and Herzegovina; 4 University Clinical Centre of the Republic of Srpska, Department of Nuclear Medicine, Banja Luka, The Republic of Srpska, Bosnia and Herzegovina; 5 University of Banja Luka, Faculty of Medicine, Banja Luka, Republic of Srpska, Bosnia and Herzegovina; 6 University Clinical Centre of the Republic of Srpska, Institute of Laboratory Diagnostics, Banja Luka, Republic of Srpska, Bosnia and Herzegovina; 7 University of Belgrade, Faculty of Pharmacy, Department of Medical Biochemistry, Belgrade

**Keywords:** cystatin C, paraoxonase 1, non-Hodgkin lymphoma, cistatin C, paraoksonaza 1, ne-Hodžkinov limfom

## Abstract

**Background:**

This pilot study aimed to assess the potential of cystatin C (Cys C) as a biomarker in non-Hodgkin lymphoma (NHL) and to explore its association with paraoxonase 1 (PON1) activity.

**Methods:**

The study included 44 patients with B-cell NHL and 44 healthy subjects. Cys C was measured using the Cobas e 801 analyser (Roche Diagnostics GmbH, Mannheim, Germany), while PON1 and TAS were measured on the ILab 300+ system (Instrumentation Laboratory, Milan, Italy).

## Abstract (sr)

**Uvod:**

Cilj ove pilot-studije bio je da se proceni potencijal cistatina C (Cys C) kao biomarkera kod ne-Hodžkinovog limfoma (NHL) i da se ispita njegova povezanost sa aktivnošću paraoksonaze 1 (PON1).

**Metode:**

U studiju su uključena 44 pacijenta sa B-ćelijskim NHL-om i 44 zdrava ispitanika. Cys C j'e meren pomoću analizatora Cobas e 801 (Roche Diagnostics GmbH, Mannheim, Nemačka), dok su PON1 i TAS određivani na sistemu ILab 300+ (Instrumentation Laboratory, Milano, Italija).

**Rezultati:**

Serumski nivo Cys C (mg/L) bio je značajno viši kod pacijenata sa NHL u poređenju sa kontrolnom grupom: 1,03 (0,88-1,24) naspram 0,83 (0,78-0,90), P&lt;0,001. Dij'agnostička vrednost Cys C, procenj'ena površinom ispod ROC krive (AUC), iznosila j'e 0,812 (95% CI: 0,732-0,902). Optimalna granična vrednost za Cys C bila je 0,870 mg/L, sa senzitivnošću od 77,1% i specifič-nošću od 75,6%. Uočena je značajna negativna korelacija između Cys C i aktivnosti PON1 (r =-0,42; P=0,010) kod pacijenata sa NHL.

**Zaključak:**

Ova studija ukazuje na potencijal Cys C kao biomarkera kod NHL. Posebno je značajno da je u multivariatnoj analizi Cys C bio dominantan nezavisni predik-tor NHL, pri čemu je svako povecanje od 1 SD u vrednosti Cys C bilo povezano sa približno 11 puta vecim izgledima za prisustvo NHL. Takođe, negativna korelacija između Cys C i PON1, prvi put identifikovana kod pacijenata sa NHL, može pružiti dragocene uvide u molekularne mehanizme koji leže u osnovi ovog maligniteta.

## Introduction

Cystatin C (Cys C) has been identified as a reliable marker of estimated glomerular filtration rate (eGFR) [1]. In the absence of significant tubular damage, this protein is reabsorbed and metabolised in the proximal tubules. Cys C has also been proposed as an early biomarker of acute kidney injury, a predictor of cardiovascular disease risk and transplant failure, and a more precise indicator of organ function in specific patient populations, including those with liver cirrhosis and oncological diseases [2]. As an inhibitor of cysteine proteases in the cathepsin family, Cys C plays a crucial role in maintaining proteolytic system balance, which may be disrupted in various pathological processes, such as during cancer development and progression [3]. In non-Hodgkin lymphoma (NHL), elevated Cys C levels and, consequently, reduced cathepsin activity may promote a pro-tumour microenvironment by limiting cathepsin-mediated antigen processing and thereby compromising immune surveillance, as well as by modulating extracellular matrix remodelling in a manner that supports lymphoma cell proliferation. Evidence from large cohort studies indicates that higher circulating Cys C concentrations are associated with increased mortality from malignancies [4].

**Figure 1:**
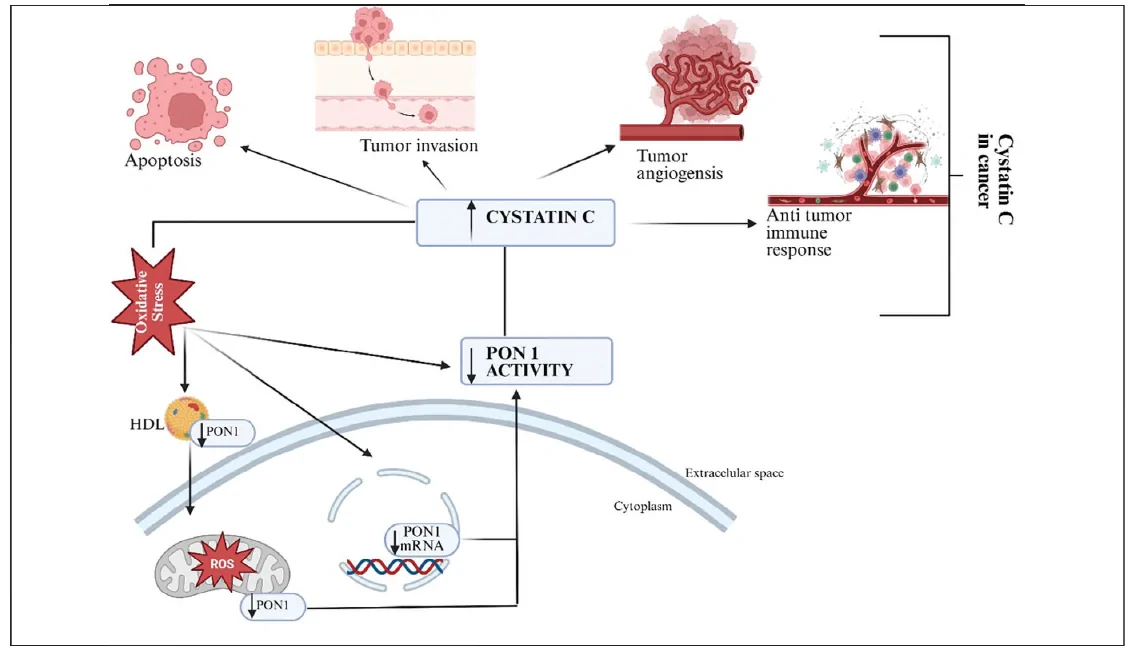
The interplay between Cys C and PON1: a hypothetical biochemical pathway.

Oxidative stress plays a significant role in the pathogenesis of NHL. A review article by Corbeanu and Gaman highlighted how reactive oxygen species (ROS) and oxidative damage contribute to the initiation and progression of lymphomas, including NHL [5]. Figure 1

Paraoxonase 1 (PON1) exhibits organophosphatase, paraoxonase, and lactonase activity, hydrolysing a wide range of substrates and providing protection against lipid oxidation [6]. It is synthesised in the liver and secreted into the plasma, where it predominantly binds to high-density lipoprotein (HDL). PON1 exerts antioxidant, antithrombotic, anti-adhesive, anti-inflammatory, and anti-apoptotic effects, as well as lipid-modifying properties [7]. It is thought that PON1 accounts for most of HDL's antioxidant capacity, as it inhibits lipoprotein peroxidation, stimulates reverse cholesterol transport, and reduces the formation of oxidised low-density lipoprotein (oxLDL) [6][7][8].

NHL represents a heterogeneous group of lymphoproliferative malignancies that frequently involve extranodal sites and exhibit greater biological unpredictability than Hodgkin lymphoma [9]. Although the aetiology of NHL has been extensively investigated, the precise cause of the disease remains unclear. Increasing attention has also been directed toward Cys C as a potential contributor to the development of lymphoproliferative disorders, given its role in regulating proteolytic activity and inflammatory responses [10][11]. The objective of this pilot study was to evaluate whether Cys C could serve as a useful biomarker in NHL and to examine its relationship with PON1 activity.

## Materials and methods

The study was conducted at the University Clinical Centre of the Republic of Srpska in Banja Luka, Bosnia and Herzegovina, in accordance with the Declaration of Helsinki. The study protocol was approved by the Ethics Committee of the University Clinical Centre of the Republic of Srpska (No. 01-19-51-2/20). All participants provided written informed consent before they participated in the study.

### Subjects

This pilot study enrolled 44 newly diagnosed patients with NHL and 44 healthy volunteers as a control group at the University Clinical Centre of the Republic of Srpska between July 2020 and April 2022. The inclusion criteria for patients were: age over 18 years, newly diagnosed B-cell NHL as the primary malignancy, and no prior history of cancer. The control group consisted of healthy volunteers who met the following criteria: age over 40 years (to match age between patients and healthy subjects adequately), both sexes, no history of malignancy or chronic disease, no acute infection within the previous three months, laboratory parameters within the normal reference range, and not pregnant or breastfeeding.

### Laboratory analyses

The patients' blood samples were obtained after overnight fasting and collected into serum tubes, then centrifuged immediately. Samples were then stored at -80°C until analysis. Cys C was measured using the Cobas e 801 analyser (Roche Diagnostics GmbH, Mannheim, Germany). Serum creatinine was quantified using standard procedures on an Alinity Abbott analyser (Abbott Laboratories, IL, USA), and the CKD-EPI creatinine equation was used to calculate eGFR. PON1 activity was assessed using paraoxone as a substrate. The conversion of paraoxone to p-nitrophenol by the hydrolytic activity of PON1 was measured at 405 nm using an ILAB 300+ analyser (Instrumentation Laboratory, Milan, Italy) according to Richter and Furlong [12]. The intra-assay coefficient of variation (CV) was 5.4%, and the inter-assay CV was 7.7%. Total antioxidant status (TAS) was determined using the method described by Erel [13]. The principle of the method is based on the reduction of the coloured 2,2'-azino-bis-(3-ethylbenzothiazoline)-6-sulfonate (ABTS)+ cation to the colourless ABTS in the presence of antioxidants in the sample. This reduction results in a decrease in the colour intensity of the ABTS solution at 660 nm, which is inversely proportional to the concentration of total antioxidants present in the sample. The intra- and inter-assay CVs for TAS were 3.05% and 4.43%, respectively.

### Statistical analysis

Statistical analyses were performed using R. Continuous variables were first assessed for normality using the Shapiro-Wilk test. Since the variables were not normally distributed, differences between the control and NHL groups were evaluated using the Wilcoxon rank-sum test. Spearman partial correlation coefficients were calculated to assess the direct, pairwise associations among Cys C, creatinine, eGFR, PON1 activity, and TAS, while adjusting for the influence of the other biomarkers. The resulting coefficients were visualised in a network graph. Independent predictors of NHL were identified by fitting a multivariable logistic regression model that included Cys C, creatinine, eGFR, PON1 activity, TAS and age as covariates to address potential confounding. Results were expressed as odds ratios (ORs) for each predictor. Finally, the discriminatory ability of Cys C was quantified by plotting a receiver operating characteristic (ROC) curve, calculating the area under the curve (AUC), and determining the optimal cut-off value using Youden's index. All tests were two-sided, and *P*-values <0.05 were considered statistically significant.

## Results

The study population consisted of 44 patients with NHL and 44 healthy subjects. Among NHL subtypes, the follicular subtype was the most common (47.73%), followed by diffuse large B-cell lymphoma (DLBCL) (31.81%) and small lymphocytic lymphoma (SLL) (9.10%). Table 1 summarises the demographic and laboratory characteristics of NHL patients and healthy controls, including age, sex, creatinine, eGFR, Cys C, PON1 activity, and TAS.

**Table 1:** Demographic and laboratory characteristics of NHL patients and healthy controls. Non-Hodgkin lymphoma, NHL; Cystatin C, Cys C; Paraoxonase 1, PON1; total antioxidant status, TAS; estimated glomerular filtration rate, eGFR; IQR; standard deviation, SD; statistically significant, P < 0.05.

Variable	NHL patients<br>(n=44)	Controls<br>(n=44)	P-value
Age, years (median, IQR)	63.5 (54-71)	58.5 (54.0-63.2)	0.065
Sex, male n (%)	22 (50)	18 (41)	0.392
Cys C, mg/L (median, IQR)	1.03 (0.88-1.24)	0.83 (0.78-0.90)	<0.001
Creatinine, μmol/L (median, IQR)	73.05 (63-80.2)	70.6 (64.9-75.7)	0.385
eGFR, mL/min/1.73 m^2^ (mean ± SD)	90.6±14	93.86±12.85	0.457
PON1 activity, U/L (median, IQR)	222 (169-521)	234 (167-452)	0.663
TAS, μmol/L (median, IQR)	998 (845.5-1139)	1175 (1092-1236)	0.003

The groups were matched for sex and age. To account for the potential confounding effect of age, we included it as a covariate in the multivariable logistic regression model. Importantly, adjusting for age did not change the association between Cys C and NHL, which remained statistically significant, indicating that the observed elevation of Cys C in NHL patients cannot be explained by age. When comparing serum biomarkers between controls and NHL patients, Cys C differed significantly (*P*<0.001), with markedly higher levels in the NHL cohort. No significant differences in creatinine levels or eGFR values were observed between patients and healthy controls. Among NHL subtypes, Cys C levels were comparable between the follicular subtype (n=21) and DLBCL (n = 14), with median values of 1.03 (0.89-1.36) and 1.0з (0.88-1.19), respectively.

In the multivariable logistic regression model including Cys C, serum creatinine, eGFR, PON1 activity, TAS, and age, Cys C and TAS emerged as statistically significant predictors of NHL (Table 2). Higher serum Cys C levels were strongly associated with increased odds of NHL (OR 11.02, 95% CI 2.6 -101.68, *P*<0.001). Although TAS was also statistically significant (OR 1.00, 95% CI 0.99-1.00, *P*=0.014), its effect was small, suggesting limited clinical relevance compared with Cys C.

**Table 2:** Multivariable logistic regression analysis of predictors of NHL. Odds ratio, OR; Cystatin C, Cys C; Paraoxonase 1, PON1; total antioxidant status TAS.

Predictor	OR	95% CI	P-value
CysC (per 1 SD)	11.02	2.65-101.68	<0.001
Creatinine	1.01	0.93-1.11	0.812
eGFR	1.07	0.99-1.15	0.069
PON1	1.00	0.99-1.00	0.963
TAS	1.00	0.99-1.00	0.014
Age (per year)	1.13	0.99-1.29	0.065

**Figure 2:**
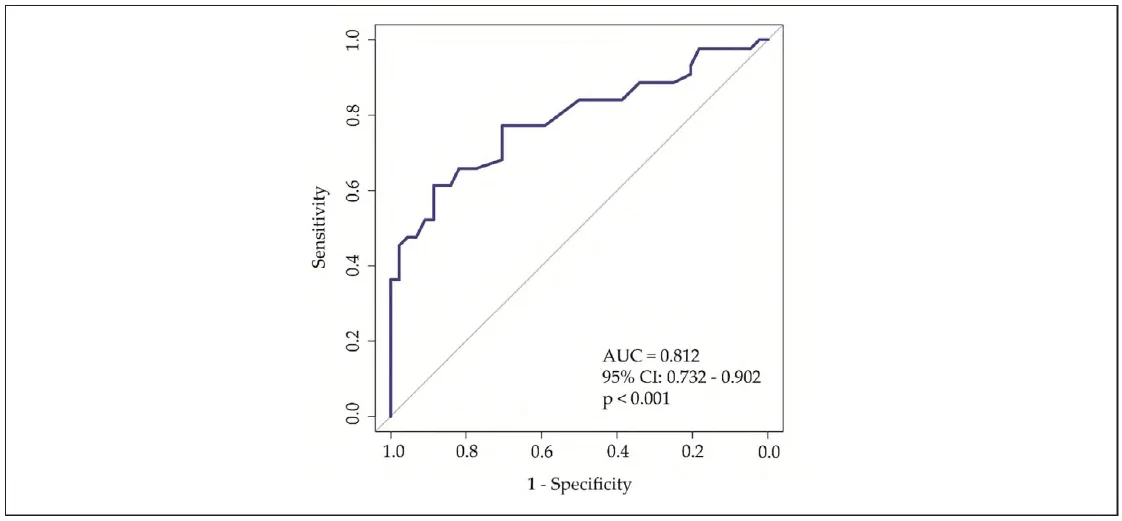
Receiver-operating characteristic (ROC) curve for Cys C in distinguishing NHL patients from healthy controls.

The receiver-operating characteristic (ROC) curve for Cys C (Figure 2) demonstrates good discriminative ability in distinguishing NHL cases from controls. The area under the curve (AUC) is 0.812 (95% CI: 0.732-0.902, *P*<0.001), indicating that Cys C has potential as a diagnostic biomarker for NHL.

At the optimal cut-off value of 0.87 mg/L, Cys C showed a sensitivity of 77.1% and a specificity of 75.6%. At this threshold, the positive likelihood ratio (LR+) was 3.16, while the negative likelihood ratio (LR-) was 0.30.

**Figure 3:**
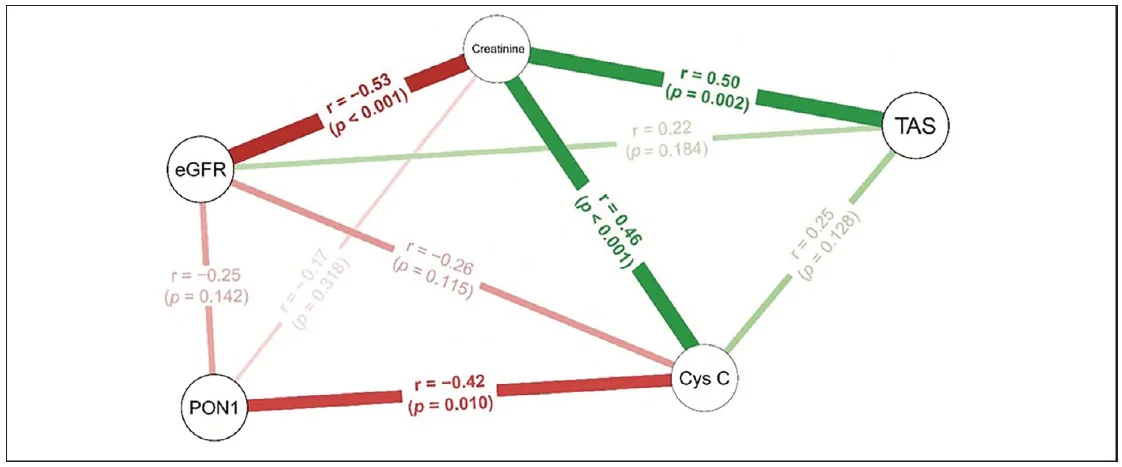
Network graph of Spearman partial correlations among biomarkers in NHL patients.

To isolate the direct monotonic relationships among biomarkers - free from confounding by the others - we calculated Spearman partial correlation coefficients and displayed them as a network graph (Figure 3).

In this network, four edges remained statistically significant: Cys C and creatinine serum exhibited a moderate positive correlation (r=0.46, *P*<0.001); creatinine and eGFR exhibited a moderate inverse correlation (r=-0.53, *P*<0.001), reflecting their well-known physiological coupling; creatinine and TAS showed moderate positive correlation (r=0.500, *P*=0.002); and Cys C and PON1 activity were inversely associated (r=-0.42, *P*=0.010), suggesting that, independent of renal and oxidative-stress measures, higher PON1 activity corresponds to lower Cys C levels. All other adjusted associations were weak and non-significant, indicating that their bivariate correlations largely reflect indirect pathways through these core relationships.

## Discussion

In this study, no significant differences in serum creatinine levels or eGFR were observed between patients with NHL and healthy controls, indicating preserved kidney function in both groups. However, a significantly higher concentration of Cys C was found in the patient group. This finding suggests that elevated Cys C levels are not the result of impaired renal function but may instead reflect the pathophysiological mechanisms of lymphoma. Based on this, Cys C may have potential as a biomarker in the context of NHL, regardless of its established role in assessing kidney function.

Because Cys C levels increase with age and may reflect renal function, we specifically adjusted our analyses for age, serum creatinine, and eGFR. Even after this adjustment, Cys C remained significantly associated with NHL, indicating that its diagnostic value cannot be explained solely by age or renal function.

Significantly higher Cys C levels in the patient group may be attributed to other factors, such as oxidative stress and inflammation, which were present in this group and have been reported previously [8]. Moreover, the previously reported increase in cathepsin S levels in this patient group [14] may partially explain the elevated Cys C concentrations, considering that Cys C serves as a natural inhibitor of cathepsin S. Additionally, the study by Hammouda et al. [15] demonstrated significantly higher serum concentrations of Cys C in patients with B-cell NHL compared with healthy controls, with the highest levels observed in patients experiencing relapse. Furthermore, our results align with those of the study by Nishio et al. [16], which suggested that oxidative stress may regulate Cys C expression via reactive oxygen species. However, it remains unclear how reactive oxygen species activate Cys C expression. Additionally, the results of this study are consistent with those of Softie A. et al. [17], who found that Cys C levels were significantly elevated in the serum of patients with aggressive NHL, with higher levels observed in advanced stages of the disease.

Although the underlying mechanisms cannot be fully elucidated from our data, increased Cys C levels in NHL may reflect upregulation of protease inhibition in response to tumour-related overexpression of cysteine cathepsins, which play roles in extracellular matrix degradation, tumour invasion, and immune evasion. Elevated Cys C could therefore represent a compensatory mechanism that counteracts cathepsin activity. In addition, higher Cys C levels may be influenced by subclinical renal impairment, systemic inflammation, or increased tumour burden, all of which have been associated with lymphoid malignancies. Our findings of elevated Cys C in NHL may indicate a link between altered protease-antiprotease balance and lymphoma pathophysiology. Importantly, in the multivariable analysis, Cys C emerged as the strongest independent predictor of disease presence. Although TAS reached statistical significance, its effect size was small, indicating that Cys C represents the primary driver of the observed association. These findings suggest that Cys C may reflect underlying malignant or inflammatory processes rather than simply serving as a marker of renal function. Further studies are warranted to elucidate the mechanisms linking Cys C with lymphomagenesis and to explore its potential utility in clinical risk stratification. ROC curve analysis demonstrated that Cys C can serve as a useful diagnostic marker for NHL. Our findings suggest that the identified cut-off value achieves a good balance between confirming and excluding the diagnosis of NHL. This issue will likely be clarified through further research aimed at identifying early and reliable biochemical markers accessible in routine clinical laboratories.

PON1 activity was lower in patients with NHL compared with healthy controls, although the difference did not reach statistical significance. It is well known that during oxidative stress in cancer, lipid peroxides are generated, and PON1 is involved in their removal [18]. In addition, TAS was significantly reduced in NHL, likely due to increased free radical production by tumour cells and surrounding tissue, which can damage lipids, proteins, and DNA. This, in turn, depletes antioxidant resources and diminishes overall antioxidant capacity. The inverse relationship between Cys C and PON1 suggests a potential link between increased inflammatory or oxidative stress and reduced enzymatic antioxidant defence. Since PON1 is associated with HDL and plays a crucial role in preventing lipid oxidation, its decreased activity may reflect impaired antioxidant protection and increased oxidative damage, which are commonly observed in malignancies, including NHL [8].

To the best of our knowledge, this study is the first to demonstrate a negative correlation between PON1 and Cys C in patients with NHL. These results are consistent with previous studies investigating the role of Cys C in oxidative stress and immune responses across various pathological conditions. For instance, a negative correlation between Cys C and superoxide dismutase, an antioxidant enzyme, was observed in patients with polycystic ovary syndrome, highlighting the potential link between Cys C and oxidative imbalance [19]. Similarly, Varga et al. [20] reported an association between Cys C and PON1 activity in hemodialysed and transplanted patients. However, they attributed this to impaired renal function in those populations [20]. Beyond its role in renal physiology, Cys C also modulates immune responses, influencing inflammatory pathways and the responses they provoke [21]. Therefore, elevated Cys C levels in NHL patients may reflect the tumour's capacity to induce inflammatory and immune responses, indirectly indicating its biological aggressiveness [22]. In addition, a negative correlation between Cys C and PON1 in NHL could be linked to apoptosis. Cys C, an inhibitor of cysteine proteases, may influence apoptotic pathways, while PON1, an antioxidant enzyme, could affect oxidative stress and the regulation of cell death [21].

The relationship between these two proteins may reflect the complex interplay between protective mechanisms and apoptotic processes in NHL. Moreover, recently published results in the same patient group showed a positive correlation between cathepsin S and small, dense HDL subfractions [14], which are considered to be rich in PON1 and possess anti-apoptotic properties. Therefore, it is not surprising that Cys C, as a natural inhibitor of cathepsin S, showed a negative correlation with PON1. Our results suggest that the relationship between Cys C and PON1 may also result from apoptosis. Further studies are needed to investigate this. Understanding the pathogenesis and progression of NHL, as well as identifying potential prognostic or therapeutic strategies, may benefit from the observed negative correlation between Cys C and PON1. While this negative correlation is an important finding, the proposed mechanisms remain hypothetical.

Furthermore, a significant positive correlation between serum creatinine and TAS was observed. This correlation may result from systemic oxidative stress affecting various metabolic pathways, including creatinine metabolism. In contrast, Cys C, a more sensitive marker of glomerular filtration, showed no correlation with TAS, suggesting that the observed association between creatinine and TAS is unlikely to reflect changes in renal function. Although primarily used as a marker of renal function, creatinine may also reflect muscle metabolism and catabolic processes, which are often more pronounced in malignant diseases [23][24]. This implies that elevated TAS levels do not necessarily indicate a protective response, but may instead serve as a marker of metabolic imbalance associated with disease progression. One possible explanation is that TAS measures the combined activity of all reducing substances present in the blood, including creatinine, glucose, bilirubin, vitamin C, urea, and others [25][26].

The main limitation of our study is the relatively small sample size, which reduces the generalizability of the results and the ability to explore differences across NHL subtypes. The cut-off value derived from the ROC curve may also be prone to overfitting in such a cohort; however, we aimed to provide an initial estimate of the diagnostic potential of Cys C.

## Conclusions

Findings from this pilot study indicate the potential of serum Cys C as a biomarker for NHL. In the multivariable logistic regression analysis, Cys C (per 1 SD increase) remained an independent predictor of NHL, with each 1 SD increase associated with approximately an 11-fold increase in the odds of disease. Additionally, partial correlation analysis revealed a negative correlation between Cys C and PON1 activity, suggesting a potential link with oxidative stress pathways in lymphoma biology. However, further studies are needed to clarify the underlying mechanisms and the significance of the interaction between Cys C and PON1.

## Dodatak

### List of abbreviations

Non-Hodgkin lymphoma, NHL;<br>cystatin C, Cys C;<br>paraoxonase 1, PON1;<br>total antioxidant status, TAS;<br>estimated glomerular filtration rate, eGFR;<br>receiver-operating characteristic, ROC;<br>area under the ROC curve, AUC;<br>confidence interval, CI;<br>interquartile range, IQR;<br>standard deviation, SD;<br>statistically significant, P<0.05.

### Funding

This work is supported by the Ministry for Scientific/Technological Development, Higher Education and Information Society, Government of the Republic of Srpska (Grant No. 1257023).

### Conflict of interest statement

All the authors declare that they have no conflict of interest in this work.
